# Limited evidence for heart rate variability as a predictor of cognitive and pathophysiological brain markers

**DOI:** 10.1177/13872877251409343

**Published:** 2025-12-30

**Authors:** Sofia Marcolini, Jaime D. Mondragón, Arie M. van Roon, Balewgizie Sileshi Tegegne, Harriëtte Riese, Rozemarijn Vliegenthart, Rudi A.J.O. Dierckx, Ronald H. Borra, Peter Paul De Deyn

**Affiliations:** 1 Department of Neurology, University Medical Center Groningen, University of Groningen, Groningen, The Netherlands; 2 The Research School of Behavioural and Cognitive Neurosciences, University of Groningen, Groningen, the Netherlands; 3 Universidad Nacional Autónoma de México, Instituto de Neurobiología, Departamento de Neurobiología Conductual y Cognitiva, Laboratorio de Psicofisiología, Querétaro, Mexico; 4 San Diego State University, Department of Psychology, Life-Span Human Senses Lab, San Diego, CA, USA; 5 Department of Vascular Medicine, University of Groningen, University Medical Center Groningen, Groningen, The Netherlands; 6 Department of Epidemiology, University of Groningen, University Medical Center Groningen, Groningen, The Netherlands; 7 Department of Psychiatry, University of Groningen, University Medical Center Groningen, Groningen, The Netherlands; 8 Department of Radiology, University Medical Center Groningen, Groningen, The Netherlands; 9 Department of Nuclear Medicine, University of Groningen, University Medical Center Groningen, Groningen, The Netherlands; 10 Laboratory of Neurochemistry and Behavior, Experimental Neurobiology Unit, University of Antwerp, Wilrijk, Antwerp, Belgium

**Keywords:** Alzheimer's disease, biomarkers, cognition disorders, dementia, health status, heart rate variability

## Abstract

**Background:**

Heart rate variability (HRV) is related to cognitive functioning and may serve as an early Alzheimer's disease biomarker.

**Objective:**

We examine whether HRV predicts cognitive and pathophysiological brain markers assessed eight and thirteen years later, independently of coronary calcification.

**Methods:**

269 cognitively unimpaired adults were selected based on their coronary artery calcification score (absent, score=0; high, score≥300), obtained from cardiac computed tomography scans (T2, 2017–2022). HRV in the time domain (root mean square of successive RR interval differences), measured at T0 (2007–2013), T1 (2014–2017), and the change between T0 and T1, was the predictor. Outcomes included cognitive measures, serum Alzheimer's disease biomarkers, and brain imaging markers obtained at T3 (2022–2023). Linear regression models were run, stratified by coronary calcification groups and adjusted for demographics, lifestyle and cardiometabolic factors.

**Results:**

Participants with high T2 calcification showed lower HRV at T0 and a positive change compared to those with absent calcification. In participants with high T2 calcification, higher HRV at T0 was associated with lower Aβ_42_/Aβ_40_ at T3, while associations with all other markers were not significant. HRV at T1 and the change were not associated with any of the outcomes.

**Conclusions:**

HRV was not associated with cognitive and brain imaging outcomes. In participants with high calcification, higher HRV measured thirteen-year earlier, typically a marker of a healthier state, was associated with a lower Aβ_42_/Aβ_40_ ratio, typically linked to Alzheimer's disease. Findings underscore the need to consider coronary calcification in research of HRV as a marker of cognitive decline.

## Introduction

Autonomic dysfunction is common in individuals with dementia and can lead to falls and syncope. The autonomic nervous system, comprising sympathetic and parasympathetic divisions, regulates primary cardiovascular functions like blood pressure and cerebral perfusion.^1^ Parasympathetic output mediated by the vagus nerve is critical for beat-to-beat control of the heart rate.^2^ Heart rate variability (HRV) is a marker of parasympathetic or vagal nerve control of the heart rhythm. Derived from electrocardiogram recordings, it measures the interval between successive heartbeats (RR intervals).^3^ Lower HRV predicts mortality,^4^ while higher HRV is linked to better cognitive and behavioral scores and is considered a marker of self-regulatory processes in individuals with neurodegenerative conditions.^5^ As an accessible and non-invasive measure, it holds promise as an early biomarker of cognitive decline.^6^

In cognitively unimpaired individuals, higher HRV is associated with better executive function, emotional regulation, and decision making.^7^ While executive functioning is the most studied cognitive domain in relation to HRV,^8^ findings on other cognitive domains remain controversial.^9^ Less research has explored the longitudinal link between cognition and HRV, but higher HRV appears to benefit later executive functioning, while its effect on episodic memory is unclear.^10^ The Neurovisceral Integration Model explains this connection through cortical integration of executive, autonomic, and emotional functions via a brain-vagus nerve feedback loop, which regulates physiological responses to environmental stimuli.^11^ Alternatively, the polyvagal theory links HRV to ventral vagal complex activity, affecting emotional regulation and social behavior.^12^

Neuroimaging studies highlight the peripheral nervous system's role in cognition and the heart-brain axis, linking HRV to brain volume, activity, and connectivity in cognitively unimpaired individuals.^13^ Lower HRV was associated with a higher burden of cerebral small vessel disease in people with diabetes but not in those without,^14^ while no correlation was found with white matter hyperintensities in a community-based study.^15^ Recent research suggests that HRV biofeedback may positively impact plasma Alzheimer's disease biomarkers,^16^ although direct studies on HRV and these biomarkers are lacking. Blood-based biomarkers like amyloid-beta_42/40_ ratio (Aβ_42_/Aβ_40_), glial fibrillary acid protein (GFAP), and neurofilament light (NfL) have shown promising for predicting and monitoring Alzheimer's disease, even in its asymptomatic stages,^17^ where lower Aβ_42_/Aβ_40_ and higher pTau181, NfL, and GFAP are linked to the disease.

Inconsistent findings on HRV's association with cognitive decline may stem from differences in cardiovascular factors impacting the underlying mechanisms. Autonomic dysfunction affects vascular health,^18^ and lower HRV predicted coronary atherosclerosis (atheromatous plaques buildup in the coronary arteries) progression.^19^ Individuals with subclinical coronary atherosclerosis, indicated by a coronary artery calcium (CAC) score higher than zero, were shown to have lower HRV compared to those with a score of zero.^18^ Overall, the reciprocal influence between autonomic and vascular systems suggests that coronary atherosclerosis may moderate the relationship between HRV and cognitive decline.^18^

To examine the link between HRV and cognitive and pathophysiological markers, we analyzed HRV at two timepoints and its change over time in relation to cognitive function, brain and white matter hyperintensities volume, and serum Alzheimer's disease biomarkers measured thirteen years later. To account for differences in coronary calcification, we conducted separate analyses for individuals with absent or high coronary calcification. We expected higher HRV to be associated with better cognitive, serum, and imaging outcomes.

## Methods

This prospective cohort study used data from Lifelines and two sub-studies, Imalife and Memolife. Lifelines is a large, multi-disciplinary cohort study that began in 2006, tracking the health and behaviors of 167,729 individuals across three generations in the North of the Netherlands. It assesses biomedical, socio-demographic, behavioral, physical, and psychological factors, with a focus on multi-morbidity and complex genetics.^20^

**Figure 1.**
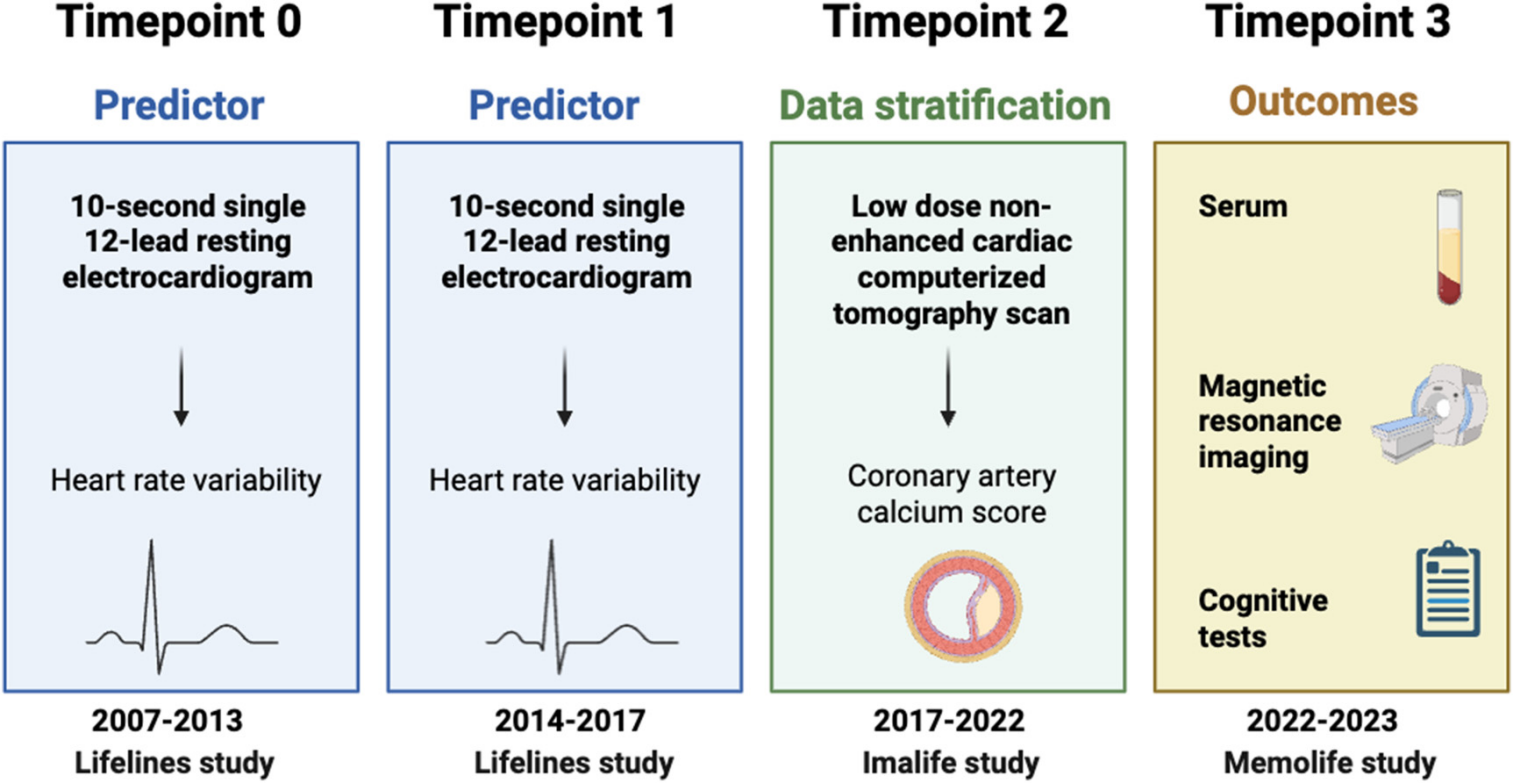
Overview data collection and timepoints. *Created with BioRender.com*.

Participants were selected based on availability of electrocardiogram data at timepoint 0 (T0) (Lifelines; 1a = 2007–2013); the 95% of these also underwent an electrocardiogram at timepoint 1 (T1) (Lifelines; 2a = 2014–2017). All participants underwent a cardiac computed tomography scan at timepoint 2 (T2) (2017–2022; Imalife) and neuropsychological, magnetic resonance imaging (MRI), and blood assessments at timepoint 3 (T3) (2022–2023; Memolife). Lifelines timepoints 1a and 2a will be referred to as timepoints 0 (T0) and 1 (T1), and the outcomes assessment (2022–2023; Memolife) as timepoint 3 (T3). Figure 1 provides an overview of the measures collected and the relative timepoints.

A total of 285 cognitively unimpaired adults from Imalife participated in Memolife. From Imalife we selected participants with a coronary artery calcification score of 0 (absent calcification) and with a score ≥ 300 (high calcification). At T3, all participants were 50 years or older, had no vascular, neurological, or psychiatric disorders two years before inclusion, and had no MRI contraindications. No additional exclusion criteria related to pre-existing medical conditions (e.g., diabetes, hypertension) were applied; therefore, participants were not selected based on these conditions. Assessments took place at the University Medical Center Groningen (UMCG). The Lifelines protocol was approved by the UMCG Medical Ethics Committee (2007/152), and the Memolife study by the UMCG Medical Ethics Review Board (NL70343.042.20, 26-11-2020). All participants provided informed consent, and procedures adhered to the Declaration of Helsinki.

**Figure 2.**
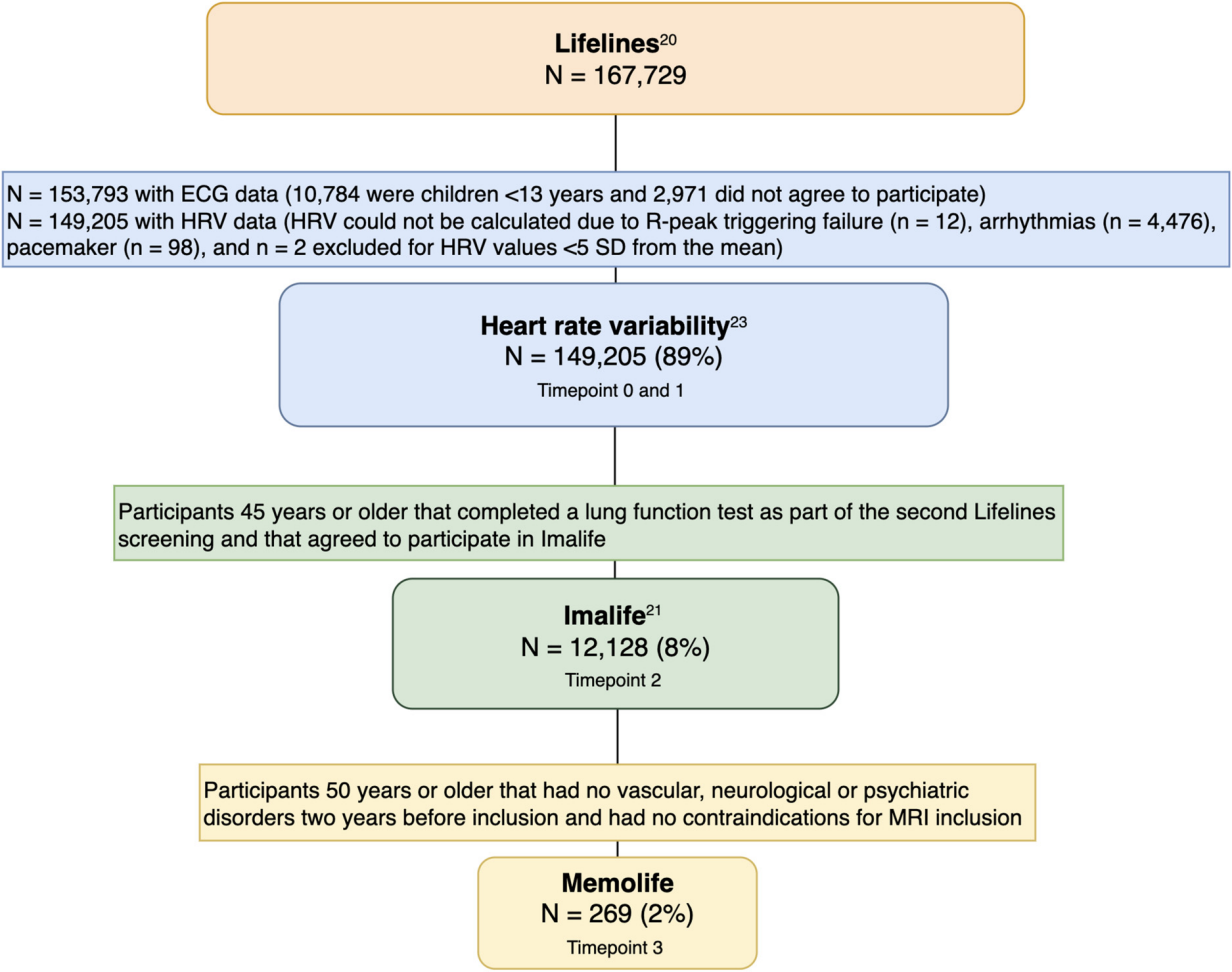
Overview studies and sample derivation.

Only participants with available data for the main exposure (HRV), stratification (Imalife), and outcomes (Memolife) were included in the analyses. Specifically, from the 167,548 participants in the Lifelines baseline cohort, 92% had available ECG data. HRV could be calculated for 97% of these participants resulting in a final analytic sample representing 89% of the initial baseline population.^23^ The Imalife study, registered with the Dutch Central Committee on Research Involving Human Subjects (https://www.toetsingonline.nl, NL58592.042.16), included 12,128 participants representative of the general Northern European population aged 45 years and older. A random subset of participants from the Imalife cohort was invited to participate in the Memolife study, aiming for a target sample size of approximately 285 individuals. Of these, 269 participants had valid HRV data and were therefore included in the final analyses (see Figure 2 for an overview of the inclusion flow and reasons for exclusion).

### Coronary artery calcification

Coronary artery calcification is a surrogate of atherosclerotic coronary disease and was used in our study to group participants and run stratified analyses. A low dose non-enhanced cardiac computed tomography scan was performed using third-generation dual-source computerized tomography (Somatom Force, Siemens Healthineers, Germany) as part of the Imalife study between September 2017 and March 2022 (T2). The cardiac computed tomography scan covered the whole heart from carina to the apex. More information on the acquisition can be found elsewhere.^21^ To quantify the total amount of CAC, the Agatston score was used,^22^ a proxy of subclinical atherosclerosis.

### Predictor assessment

*Electrocardiogram.* A 10-second single 12-lead resting electrocardiogram was recorded using the Cardioperfect software (Welch Allyn DT100 recorder, Welch Allyn, Skaneateles Falls, NY). To assess HRV, electrocardiogram signals were obtained from the Lifelines electrocardiogram database using a specialized in-house software, which automatically computes HRV indices. The detailed protocol and reasons for excluding some of the electrocardiogram recordings have been described elsewhere.^23^ Electrocardiogram signals were carefully reviewed by Lifelines cardiologists to determine their suitability for analysis and electrocardiogram recordings displaying arrhythmia, pacemaker usage, or triggering failures were excluded from the analysis. For this analysis, HRV in the time domain was used calculating the log-transformed root mean square of successive RR interval differences (RMSSD); throughout the manuscript we refer to HRV indicating this measure. The ECG recordings followed the standard diagnostic cardiology protocol, which has a 10-second duration. While 24-hour recordings provide a more comprehensive assessment of autonomic function, shorter recordings are a practical necessity in large cohort studies. The RMSSD metric is well-suited for short-term analysis as it reflects parasympathetic (vagal) activity and has been validated against longer recordings.^24,25^ For this reason, only the RMSSD metric was used, as the standard deviation of NN intervals (SDNN) and frequency-domain measures cannot be reliably estimated from such short recordings. The ECG data were recorded at a sampling frequency of 600 Hz, as detailed in Tegegne et al. (2018).^23^ At this sampling rate, the error variance for RMSSD estimation is less than 0.3 ms^2^ (see Appendix A in Greaves-Lord et al., 2010).^26^ To assess HRV change, the raw difference between data collected at T0 and at T1 (HRV T1 – HRV T0) was calculated.

### Outcomes assessment

*Cognition.* Neuropsychological assessments were conducted according to established protocols. Cognitive tests were administered in the following order: Mini Mental State Examination (MMSE),^27^ 15 Word-Auditory Verbal Learning Test (AVLT) immediate recall and delayed recall and recognition after twenty minutes,^28^ Trail Making Test (TMT) A and B,^29^ 9-hole Purdue pegboard test,^30^ Letter Digit Substitution Test (LDST) (90 seconds),^31^ Visual Association Test (VAT),^32^ Digit Span (forward and backward),^33^ Stroop Colour Word Test (SCWT),^34^ Verbal Word Fluency Test (60 seconds, animals).^35^

Z-scores were calculated independently for each test by standardizing individual scores against the mean and standard deviation. A standardized composite score, known as the g-factor, was derived using principal component analysis to measure overall cognitive ability.^36^ The g-factor was the first unrotated component of principal component analysis and explained 50.33% of the variance. Subsequently, cognitive domain scores were computed for memory (word learning test immediate and delayed recall), executive function (Stroop interference task, verbal fluency test, and letter-digit substitution task, with equal weighting), information processing (Stroop reading and color naming task, and letter-digit substitution task, with equal weighting), and motor function (Purdue pegboard test).

### Serum Alzheimer's disease biomarkers

Blood samples were collected at the blood withdrawal center of the University Medical Centre Groningen and preserved at minus 80°C. Analysis of the Alzheimer's disease biomarkers was carried out at the Amsterdam University Medical Centers, Department of Laboratory Medicine, Neurochemistry Laboratory. The following biomarkers were measured: Aβ42/Aβ40 ratio, pTau18, NfL, GFAP. All measurements were performed on a single molecule array (Simoa) HD-x analyzer platform, with the Neurology 4-plex E kit (Quanterix, USA) in monoplo and the P-tau181 V2.1 kit (Quanterix) in duplo according to manufacturer's instructions. Prior to measurement, samples were shortly thawed at room temperature and centrifuged for 10 min at 10,000 xg.

### Brain magnetic resonance imaging

For 238 participants, brain magnetic resonance imaging data on a 3.0 Tesla scanner (Prisma, Siemens; software syngo MR E11) was acquired, using a head/neck 64 head coil. Volumetric T1-weighted, T2-weighted, T2-weighted*, FLAIR images were captured employing a 3D magnetization prepared-rapid gradient echo (MP-RAGE) sequence. Specifically, structural imaging parameters were the following: T1-weighted MPRAGE: TR 2300 ms, TE 2.26 ms, TI 900 ms, FA 8 degrees, FOV (read) 256 mm, voxel size 1.0 × 1.0 × 1.0 mm, Bandwidth 200 Hz/pixel, acquisition time 5:20. T2-FLAIR: TR 5000 ms, TE 392 ms, TI 1800 ms, FOV (read) 256 mm, voxel size 1.0 × 1.0 × 1.0 mm Bandwidth 592 Hz/Px, acquisition time 5:12. Brain volumes (medial temporal lobe, hippocampus, and total brain volume) and total white matter hyperintensity (WMH) volumes were assessed using the fully-automated cNeuro image quantification tool developed by Combinostics in Finland.^37^ This tool divides T1 images into 133 brain regions using a multi-atlas method derived from 79 manually segmented atlases.^38^ White matter hyperintensities volume segmentation was conducted on FLAIR images using a multi-stage method based on the Expectation-Maximization algorithm.^39^ All volumes were adjusted for intracranial volume.^40^

### Covariates

Data on education level (low, middle, high), smoking habit (never, former, current, recent starter), alcohol consumption, and diabetes presence were assessed using participants’ responses on self-administered questionnaires. Body mass index was calculated using body weight and height (weight (kg)/height^2^ (m)), and alcohol consumption was assessed as average consumption days per month.

### Statistical analysis

All analyses were run in R studio (2022.02.0.443). Study characteristics were reported as percentage (%) or means with standard deviation (SD). A Welch Two Sample T-Test was performed to investigate differences in HRV among coronary artery calcification groups. Linear regression models were run to determine the associations between HRV (at T0, T1, and change) and the 10 outcome variables: four cognition (memory, executive functioning, information processing, motor function), four blood biomarkers (Aβ_42_/Aβ_40_, pTau181, NfL, GFAP), and two brain (total brain volume and total WMH volume) variables. The WMH variable was log-transformed due to its skewed distribution. Models were run separately in the absent and high calcification groups. Models were run for each outcome variable: 1) adjusted for age, level of education, sex, time interval between assessments (T0 and T3; T1 and T3; and T0 and T1); 2) additionally adjusting for lifestyle and cardiometabolic covariates measured at T0 and specifically, smoking habit, systolic and diastolic blood pressure, body mass index, alcohol consumption, and diabetes presence. Sample sizes slightly differed among models due to differences in outcomes availability and for missingness of some of the covariates. All models were corrected for multiple comparisons setting a false discovery rate (FDR) at 5% using the Benjamini-Hochberg method and using a 2-tailed hypothesis tests with α = 0.05. For MRI measures, missing data primarily resulted from participants’ withdrawal (specifically for death, sickness or unspecified reasons) or from inability to complete the scan due to claustrophobia.

## Results

Two hundred and sixty-nine subjects met inclusion criteria and had available data at T0 and T3; they had a mean age of 55.49 ± 9.30 and were mostly men (58.74%). Sample characteristics for data at both T0 and T1 are reported in Table 1. 154 participants had a calcification score of 0 and 131 a score ≥ 300. Sample characteristics per group are reported in Supplemental Table 1.

**Table 1.** Sample characteristics.

Characteristics	Timepoint 0 (n = 269)	Timepoint 1 (n = 256)
Age (years)	55.49 ± 9.30	60.14 ± 8.63
Women	111 (41.26%)	107 (41.80%)
Education level		
Low	109 (40.52%)	101 (39.45%)
Middle	83 (30.86%)	80 (31.25%)
High	77 (28.26%)	75 (28.30%)
Smoking habit		
Never	91 (33.83%)	87 (33.98%)
Former	138 (51.30%)	135 (52.73%)
Current	35 (13.01%)	30 (11.72%)
Recent starter	<10 (0.37%)	0 (0%)
Systolic blood pressure (mmHG)	132.8 ± 15.85	132.7 ± 16.04
Diastolic blood pressure (mmHG)	75.59 ± 9.63	75.66 ± 9.71
Body mass index (kg/m^2^)	26.32 ± 3.31	26.29 ± 1.19
Alcohol (days per month)	4.16 ± 2.21	2.30 ± 1.78
Diabetes presence	12 (4.46%)	< 10 (2.34%)
RMSSD (ln) (ms)	3.06 ± 0.69	3.02 ± 0.70
Time interval T0 and T3 (years)	12.59 ± 1.07	
Time interval T1 and T3 (years)		7.89 ± 0.52

Mean ± SD or N (%). Data missing at T0: 4 smoking, 1 systolic blood pressure, 1 diastolic blood pressure, 8 alcohol, 9 diabetes. Data missing at T1: 4 smoking, 38 alcohol, 17 diabetes. RMSSD: root mean squared of successive RR interval differences; ln: logarithm.

### Associations between heart rate variability and outcomes in absent and high coronary artery calcification groups

Participants with high coronary artery calcification showed lower HRV at T0 (*p* = 0.001) and a positive change in HRV from T0 to T1 (*p* = 0.040) (Supplemental Table 1).

Regression models showed significant associations between higher HRV at T0 and lower serum Aβ_42_/Aβ_40_ ratio (β = -0.30; 95% CI = -0.47, -0.13) only in the high coronary artery calcification group (Table 2). These associations remained significant when correcting for additional confounders (Supplemental Table 2). HRV at T1 and the change between T0 and T1 were both not associated with any of the outcomes at T3, in both the absent and high coronary artery calcification group (*p* > 0.05) (Table 3 and Supplemental Table 3).

**Table 2.** Associations between HRV at T0 and outcomes at T3 in absent and high CAC groups.

	ABSENT CAC			HIGH CAC			
Outcomes	*n*	β Coefficient (95% CI)	*p*	*n*	β Coefficient (95% CI)	*p*	*p-*FDR
Memory	146	−0.01 (−0.16, 0.14)	0.87	120	0.03 (−0.13, 0.19)	0.70	0.82
Executive functioning	144	0.06 (−0.06, 0.18)	0.30	116	0.05 (−0.08, 0.18)	0.46	0.82
Information processing	146	0.06 (−0.07, 0.18)	0.35	117	−0.02 (−0.15, 0.11)	0.80	0.82
Manual dexterity	147	−0.04 (−0.18, 0.10)	0.55	118	0.02 (−0.15, 0.19)	0.81	0.82
General cognition	144	0.08 (−0.05, 0.21)	0.22	114	0.05 (−0.11, 0.20)	0.57	0.82
Total brain volume	80	0.02 (−0.23, 0.27)	0.87	92	−0.04 (−0.23, 0.15)	0.66	0.82
Hippocampus volume	80	0.07 (−1.17, 0.31)	0.56	92	−0.05 (−0.15, 0.24)	0.64	0.82
MTL total volume	80	0.04 (−0.22, 0.31)	0.75	92	−0.03 (−0.22, 0.17)	0.80	0.82
Total WMH volume	80	0.12 (−0.09, 0.33)	0.25	90	0.03 (−0.20, 0.25)	0.82	0.82
Aβ_42_/Aβ_40_	145	−0.06 (−0.25, 0.12)	0.50	118	−0.30 (−0.47, −0.13)	0.00*	0.01*
pTau181	143	−0.04 (−0.19, 0.12)	0.64	116	0.06 (−0.15, 0.26)	0.58	0.82
NfL	145	−0.07 (−0.21, 0.07)	0.34	118	−0.02 (−0.20, 0.15)	0.78	0.82
GFAP	145	−0.04 (−0.21, 0.14)	0.66	118	−0.03 (−0.16, 0.10)	0.66	0.82

Models are corrected for age at T0, sex, education, and time interval between T0 and T3. All analyses used z-transformed scores for all predictors and outcome variables. CAC: coronary artery calcium; CI: confidence intervals; GFAP: glial fibrillary acidic protein; NfL: neurofilament-light; FDR: false discovery rate; MTL: medial temporal lobe; pTau: phosphorylated tau; WMH: white matter hyperintensity. *p-*value* < 0.05.

**Table 3.** Associations between HRV at T1 and outcomes at T3 in absent and high CAC groups.

	ABSENT CAC			HIGH CAC		
Outcomes	*n*	β Coefficient (95% CI)	*p*	*n*	β Coefficient (95% CI)	*p*
Memory	141	−0.09 (−0.24, 0.05)	0.21	112	−0.06 (−0.22, 0.11)	0.50
Executive functioning	139	0.04 (−0.07, 0.15)	0.46	108	−0.01 (−0.14, 0.12)	0.88
Information processing	141	0.00 (−0.12, 0.13)	0.95	109	0.03 (−0.11, 0.17)	0.69
Manual dexterity	142	0.03 (−0.10, 0.17)	0.61	110	0.07 (−0.10, 0.24)	0.43
General cognition	139	0.02 (−0.11, 0.14)	0.82	106	−0.06 (−0.22, 0.11)	0.50
Total brain volume	121	0.10 (−0.09, 0.30)	0.28	86	0.03 (−0.17, 0.23)	0.77
Hippocampus volume	121	−0.04 (−0.24, 0.15)	0.66	86	0.09 (−0.12, 0.29)	0.41
MTL total volume	121	0.04 (−0.16, 0.24)	0.71	86	0.04 (−0.17, 0.24)	0.71
Total WMH volume	120	0.04 (−0.14, 0.22)	0.63	84	−0.12 (−0.35, 0.11)	0.31
Aβ_42_/Aβ_40_	140	0.05 (−0.14, 0.23)	0.61	110	−0.16 (−0.34, 0.03)	0.09
pTau181	138	−0.10 (−0.25, 0.05)	0.18	108	0.18 (−0.03, 0.38)	0.09
NfL	140	0.01 (−0.13, 0.16)	0.85	110	0.02 (−0.16, 0.20)	0.80
GFAP	140	0.02 (−0.13, 0.16)	0.83	110	0.08 (−0.05, 0.22)	0.22

Models are corrected for age at T0, sex, education, and time interval between T1 and T3. All analyses used z-transformed scores for all predictors and outcome variables. CAC: coronary artery calcium; CI: confidence intervals; GFAP: glial fibrillary acidic protein; NfL: neurofilament-light; FDR: false discovery rate; MTL: medial temporal lobe; pTau: phosphorylated tau; WMH: white matter hyperintensity.

## Discussion

The associations between HRV assessed at two timepoints and its change over time, with cognitive, brain, and serum outcomes measured approximately eight and thirteen years later were investigated. Our findings suggest that higher HRV measured approximately thirteen years earlier was linked to decreased serum Aβ_42_/Aβ_40_ ratio in cognitively unimpaired individuals with high coronary calcification, even after adjusting for demographic, lifestyle and cardiometabolic factors. This result contradicts our hypothesis, as higher HRV is typically associated with better health, while a lower Aβ_42_/Aβ_40_ ratio is linked to Alzheimer's disease. No association was found in individuals without coronary calcification. Additionally, we found no associations between HRV and cognitive outcomes, other Alzheimer's disease biomarkers, or volumetric brain measures.

### Cognitive, brain, and serum outcomes

A previous study of a multi-ethnic cohort of American adults reported that higher HRV predicted better cognitive outcomes ten years later when measured by the standard deviation of normal-to-normal intervals, but not by the root mean square of successive differences, the metric used in our study.^41^ In their design, differently from our study, their second HRV measurement corresponded to the cognitive measurement timepoint. Similarly to our study, they found no association between the change in HRV and cognition. Research on HRV and memory remains scarce and inconsistent,^10^ with some studies suggesting that greater sympathetic response predicts episodic memory decline.^10^

We also found no associations between HRV and volumetric brain measures, possibly because our sample was still cognitively healthy and perhaps too young. If we hypothesize that HRV influences cerebral perfusion first, and consequently brain volume and WMH volume, it might have been too early to detect associations with structural brain measures. Lower HRV, measured as the standard deviation of normal-to-normal intervals, was associated with increasing total burden of cerebral small vessel disease and each small vessel disease marker in a diabetic group, but not in a non-diabetic group.^14^ Similarly, higher total burden of small vessel disease and WMH were associated with lower HRV in a study using a stroke-registry sample.^42^ These findings might suggest that relationships with direct brain measures are more evident in clinical populations with a higher risk of cardiovascular and cerebrovascular disease, such as diabetes or stroke.

The lack of previous studies examining the relationship between HRV and Alzheimer's disease biomarkers makes it difficult to compare our results on the relationship between HRV and Aβ_42_/Aβ_40_ ratio. One study found that a four-week HRV biofeedback intervention, involving slow-paced breathing, affected plasma Αβ_40_, Αβ_42_, total tau, and phosphorylated tau levels.^16^ The largest effect sizes were found in plasma Aβ_40_ and Aβ_42_ change. They concluded that their study provided support for a causal role on autonomic activity in modulating plasma-Alzheimer's disease biomarkers. Our findings suggest that higher HRV measured approximately thirteen years earlier was linked to decreased serum Aβ_42_/Aβ_40_ ratio. Although this result contradicts our hypothesis, it could be explained by the possibility that amyloid deposition triggers a neuroinflammatory response, which in turn affects autonomic function and increases HRV, potentially acting as a compensatory mechanism to amyloid accumulation.^16^

While serum Alzheimer's disease biomarkers were shown to be valuable prognostic and or monitoring tools in cognitively unimpaired elderly,^17^ assessing these associations also using cerebrospinal fluid and positron emission tomography data is needed. A relationship between HRV and Aβ levels may be explained by the role of the autonomic nervous system in blood pressure regulation and other cardiovascular functions, which in turn affects brain perfusion. The noradrenergic system may also be involved considering that acetylcholine is the primary neurotransmitter involved in the parasympathetic system and that the cholinergic system has also been shown to be dysfunctional in dementia. Additionally, individuals with a family history of cognitive decline and subjective memory complaints exhibited expected vagal tone increases under stress only if they were amyloid-negative, highlighting potential autonomic differences in Alzheimer's disease risk groups.^43^

### The role of coronary calcification

Stratifying for coronary artery calcification provided further insights into biomarker-related mechanisms of cognitive decline. Individuals with high coronary artery calcification had lower HRV at baseline. In accordance with our findings, in Hoshi et al. (2023),^18^ participants with subclinical atherosclerosis, measured by a CAC score higher than zero, had lower HRV compared to those with score zero. In a large sample of older adults at risk of cardiovascular disease, lower HRV was linked to worse processing speed and reaction time, as well as to a steeper decline in processing speed after about three years.^44^ Furthermore, subclinical atherosclerosis, as measured by carotid intima-media thickness, was identified as a mediator in the relationship between HRV and executive function.^45^ While our finding that higher HRV was negatively associated with Aβ measures in individuals with high atherosclerotic burden contradicts our hypothesis, it is notable that this association emerged in those with greater cardiovascular risk. Recent work by Alaka et al. (2024)^46^ demonstrated that incorporating resting heart rate (RHR) into the CAIDE dementia risk model significantly improved dementia risk prediction across diverse populations. This highlights the growing recognition that autonomic and cardiovascular markers, including RHR and HRV, may add prognostic value to traditional dementia risk models. Our results only partially complement this perspective by suggesting that long-term autonomic regulation, indexed by HRV, could also relate to Alzheimer's disease-related biomarkers, particularly in individuals with higher cardiovascular burden. Although, our findings should be interpreted as preliminary evidence rather than conclusive proof of a mechanistic link between HRV and amyloid pathology. Replication in independent cohorts with longitudinal autonomic and neuroimaging data, as well as experimental studies probing causal pathways, will be essential to clarify whether the observed association represents a true biological relationship or reflects statistical or contextual factors specific to this cohort.

### Limitations and future directions

Our sample included only cognitively unimpaired individuals, potentially excluding those who later developed neurological disorders and potentially removing cases where HRV associations may be stronger. Due to the absence of data on withdrawn participants, attrition bias remains a concern. Also, since our participants were all of European descent, generalizability to other populations is limited.

Another potential limitation of this study is the multi-step inclusion process, which may have introduced selection bias. Participants who agreed to take part in the Imalife and Memolife sub-studies are likely to represent a more health-conscious and research-engaged subset of the original Lifelines cohort. Consequently, the final sample may not be fully representative of the general population, and this should be considered when interpreting the findings.

For future studies, given that lower HRV has been associated with various psychopathologies, including major depressive disorder, panic disorder, and posttraumatic stress disorder,^47^ HRV represents a promising measure for investigating its relationship with behavioral symptoms of dementia. Additionally, while we focused on discussing results in terms of high and low HRV, an ideal range, instead of specifically high or low values has been proposed as a target for biofeedback interventions.^47^ Moreover, dynamic HRV measurements may provide a more sensitive assessment of autonomic function.^8^ Also, while using a 10-s HRV measure may be considered disadvantageous compared to 24-h monitoring, this measure is more feasible in large cohorts and is widely used in the clinic.^48^ Ultra-short recordings, have been shown to reliably estimate the RMSSD, which is relatively stable over short durations and less affected by recording length than frequency-domain measures.^24^ Several peer-reviewed studies have validated the use of 10-s ECGs for estimating RMSSD in both epidemiological and clinical cohorts.^25,49^ This approach facilitates the integration of HRV metrics into studies involving imaging, biomarker analysis, and other time-sensitive assessments, as was the case in our study. Although, while single 10-s resting ECG is feasible for large-scale studies and RMSSD is a validated metric for short recordings, it does not capture the full spectrum of autonomic function, such as circadian rhythms or responses to stressors, that a 24-h measurement would provide. This could lead to a less comprehensive characterization of an individual's autonomic state.

As sex differences were previously found in HRV, with women having higher scores,^23^ possibly due to greater parasympathetic cardiovascular modulation or hormonal differences like estrogen and oxytocin,^50^ future studies should also consider examining these results separately in men and women, which was not possible in our sample due to the small sample size. Future studies should aim to incorporate additional potential confounders such as medication use (e.g., beta-blockers), atrial fibrillation, lipid profiles, inflammatory markers, and physical activity. Although physical activity data were available in the larger Lifelines cohort, they were not aligned with the relevant measurement time points in the present analyses. Including these factors in future work may provide a more comprehensive understanding of the observed associations. Lastly, E-values could be explored as an informative way to assess robustness to unmeasured confounding.

### Conclusion

Overall, our findings suggest that in adults with a high coronary calcification, higher HRV, generally indicative of a healthier state, was linked to lower serum Aβ_42_/Aβ_40_ ratio levels at a thirteen-year follow-up. The unexpected direction of this association warrants cautious interpretation and should be contextualized within the broader literature on the predictive value of serum Alzheimer's disease biomarkers. These results underscore the importance of considering coronary calcification when exploring the relationship between higher HRV and cognitive decline, highlighting a potential interplay between cardiovascular health and Alzheimer's disease pathology.

## Supplemental Material

sj-docx-1-alz-10.1177_13872877251409343 - Supplemental material Supplemental material, sj-docx-1-alz-10.1177_13872877251409343
