# Supplementary material for: Limited evidence for heart rate variability as a predictor of cognitive and pathophysiological brain markers
Source: J Alzheimers Dis. 2025 Dec 30;109(4):1723–32. doi: 10.1177/13872877251409343 (PMC13487330; doi:10.1177/13872877251409343)
Supplement: Supplementary file 1 [file sj-docx-1-alz-10.1177_13872877251409343.docx]

**Supplemental Material**

**Limited evidence for heart rate variability as a predictor of cognitive and pathophysiological brain markers**

**Supplemental Table 1.** Sample characteristics per CAC group

| **Characteristics** | **ABSENT CAC**  ***n = 148*** | **HIGH CAC**  ***n = 121*** | ***p*** |
| --- | --- | --- | --- |
| **Age (years)** | 54.27 (9.25) | 56.99 (9.18) | 0.02 |
| **Women** | 81 (54.73%) | 30 (24.79%) | **<0.001**** |
| **Education level** |  |  | 0.29 |
| Low | 56 (37.84%) | 53 (43.80%) |  |
| Middle | 74 (34.46%) | 31 (25.62%) |  |
| High | 16 (27.03%) | 36 (29.75%) |  |
| **Smoking habit** |  |  | 0.31 |
| Never | 55 (37.16%) | 36 (29.75%) |  |
| Former | 74 (50%) | 64 (52.89%) |  |
| Current | 16 (10.81%) | 19 (15.70%) |  |
| Recent starter | <10 (0%) | <10 (0.83%) |  |
| **Systolic blood pressure (mmHG)** | 129.80 (14.87) | 136.4 (16.30) | **<0.001**** |
| **Diastolic blood pressure (mmHG)** | 73.28 (8.70) | 78.42 (9.99) | **<0.001**** |
| **Body mass index (kg/m²)** | 25.8 (3.16) | 26.95 (3.39) | **0.01**** |
| **Alcohol (days per month)** | 4.08 (2.27) | 4.25 (2.15) | 0.56 |
| **Diabetes presence** | <10 (2.03 %) | <10 (7.44%) | 0.07 |
| **RMSSD T0, ln** | 0.18 | -0.22 | **0.00**** |
| **RMSSD T1, ln*** | 0.06 | -0.08 | 0.23 |
| **RMSSD change, ln*** | -0.12 | 0.14 | **0.04**** |

Data at T0. Mean (SD) or N (%). CAC: coronary artery calcium; ln: logarithm; RMSSD: root mean squared of successive RR interval differences. A Welch Two Sample T-Test was performed. Among women, 81 have absent CAC and 30 high CAC. Among men we have 67 that have absent CAC and 91 with high CAC. *Absent CAC n = 143; High CAC n = 113

**Supplemental Table 2.** Associations between HRV at T0 and outcomes at T3 in the high CAC group additionally adjusting for lifestyle and cardiometabolic confounders

|  | **HIGH CAC** | **Models adjusted by additional confounders** |  |  |
| --- | --- | --- | --- | --- |
| **Outcomes** | ***n*** | **β Coefficient (95% CI)** | ***p*** | ***p* FDR** |
| **Aβ_42_/Aβ_40_** | 113 | -0.30 (-0.44, -0.09) | **0.004**** | **0.01**** |
| **pTau181** | 111 | 0.12 (-0.08, 0.32) | 0.229 | 0.34 |
| **NfL** | 113 | 0.01 (-0.16, 0.17) | 0.951 | 0.95 |
| **GFAP** | 113 | -0.05 (-0.18, 0.08) | 0.464 | 0.56 |

Models are adjusted for age at T0, sex, education, time interval between T0 and T3, smoking T0, systolic and diastolic blood pressure T0, BMI T0, alcohol T0, diabetes presence T0. CAC: coronary artery calcium; GFAP: glial fibrillary acidic protein; NfL: neurofilament-light; FDR: false discovery rate; pTau: phosphorylated tau.

**Supplemental Table 3.** Associations between change in HRV and outcomes at T3 in absent and high CAC

|  | **ABSENT CAC** |  |  | **HIGH CAC** |  |  |
| --- | --- | --- | --- | --- | --- | --- |
| **Outcomes** | ***n*** | **β Coefficient**  **(95% CI)** | ***p*** | ***n*** | **β Coefficient**  **(95% CI)** | ***p*** |
| **Memory** | 141 | -0.09 (-0.23, 0.06) | 0.25 | 112 | -0.08 (-0.24, 0.09) | 0.35 |
| **Executive functioning** | 139 | -0.02 (-0.13, 0.09) | 0.73 | 108 | -0.06 (-0.20, 0.08) | 0.39 |
| **Information processing** | 141 | -0.05 (-0.17, 0.07) | 0.38 | 109 | 0.05 (-0.10, 0.19) | 0.52 |
| **Manual dexterity** | 142 | 0.08 (-0.05, 0.21) | 0.23 | 110 | 0.10 (-0.08, 0.27) | 0.27 |
| **General cognition** | 139 | -0.07 (-0.20, 0.06) | 0.27 | 106 | -0.10 (-0.27, 0.07) | 0.26 |
| **Total brain volume** | 118 | -0.03 (-0.23, 0.16) | 0.74 | 86 | 0.07 (-0.13, 0.27) | 0.51 |
| **Hippocampus volume** | 118 | -0.11 (-0.31, 0.09) | 0.27 | 86 | -0.06 (-0.14, 0.25) | 0.58 |
| **MTL total volume** | 118 | -0.09 (-0.29, 0.11) | 0.38 | 86 | -0.08 (-0.11, 0.28) | 0.40 |
| **Total WMH volume** | 117 | 0.04 (-0.14, 0.22) | 0.66 | 84 | -0.21 (-0.44, 0.03) | 0.09 |
| **Aβ_42_/Aβ_40_** | 140 | 0.10 (-0.08, 0.28) | 0.26 | 110 | 0.15 (-0.04, 0.34) | 0.12 |
| **pTau181** | 138 | -0.06 (-0.21, 0.09) | 0.42 | 108 | 0.10 (-0.11, 0.31) | 0.34 |
| **NfL** | 140 | 0.07 (-0.06, 0.21) | 0.29 | 110 | 0.07 (-0.12, 0.25) | 0.48 |
| **GFAP** | 140 | 0.04 (-0.11, 0.18) | 0.62 | 110 | 0.10 (-0.04, 0.24) | 0.16 |

Models are corrected for age at T0, sex, education, and time interval between T0 and T3. All analyses used z-transformed scores for all predictors and outcome variables. CAC: coronary artery calcium; CI: confidence intervals; GFAP: glial fibrillary acidic protein; NfL: neurofilament-light; FDR: false discovery rate; MTL: medial temporal lobe; pTau: phosphorylated tau; WMH: white matter hyperintensity.
